# Secondary Dengue Infection Presenting With Kawasaki Disease–Like Features: A Diagnostic Dilemma in a 12‐Year‐Old Girl

**DOI:** 10.1002/ccr3.72672

**Published:** 2026-05-10

**Authors:** Saad Bin Toufeeq, Ammara Arshad, Nabeel Ahmad, Ali Bin Toufeeq, Ahmad Sama, Neykzad Wadeer

**Affiliations:** ^1^ Lahore General Hospital Azra Naheed Medical College Lahore Pakistan; ^2^ Lahore General Hospital Ameer‐Ud‐Din Medical College Lahore Pakistan; ^3^ Lahore General Hospital International School of Medicine/International University of Kyrgyzstan Bishkek Kyrgyzstan; ^4^ CURE International Hospital Kabul Afghanistan

**Keywords:** dengue fever, immunoglobulins, Kawasaki disease, virology

## Abstract

In dengue‐endemic regions, prolonged fever with mucocutaneous findings may represent classical Kawasaki disease (KD), dengue‐associated KD‐like inflammation, or true concomitant disease. When KD cannot be confidently excluded, timely IVIG administration may be justified to prevent coronary complications. Structured follow‐up including serial echocardiography remains essential.

## 1 Introduction

Dengue fever is an acute viral illness which is caused by the dengue virus, a single stranded RNA virus. *Aedes aegypti* and *Aedes albopictus* are the transmitters responsible for its transmission. It is endemic globally; mostly distributed in the Eastern Mediterranean, Southeast Asia, Africa, the Western Pacific, and South America [1]. It is characterized by fever, systemic inflammation, and a spectrum of clinical features that can overlap with other febrile syndromes. In some patients especially children, dengue may present with mucocutaneous changes, lymphadenopathy, and laboratory abnormalities that resemble Kawasaki disease making it a diagnostic pitfall for clinicians. Dengue infection has been reported to either mimic Kawasaki disease (KD), trigger KD‐like inflammatory syndromes, or rarely occur concomitantly with true KD. Dengue virus can trigger inflammation, chemokine release, and endothelial cell changes, potentially causing arteritis similar to that seen in Kawasaki Disease [2]; this furthermore makes early diagnosis challenging when specific serological confirmation is still pending.

Understanding scenarios where dengue mimics KD's clinical features is significant, as it helps clinicians avoid misdiagnosis and prevents unnecessary delays in appropriate treatment. It also highlights the need for careful interpretation of clinical findings [3].

We report a rare case of secondary dengue infection in a 12‐year‐old girl who fulfilled clinical criteria for classical KD and responded to IVIG prior to confirmatory dengue serology. The subsequent laboratory evolution consistent with dengue created significant diagnostic uncertainty between true KD and dengue‐associated KD‐like inflammation. This case highlights the therapeutic and diagnostic challenges encountered in dengue‐endemic settings.

## 2 Case Presentation

A 12 year old Pakistani girl presented to the Pediatrics Department on 22 October 2025 with a 7 day history of continuous, high‐grade fever, which began on 15 October 2025. Fever was sudden in onset, persistent, and associated with rigors and chills. She denied symptoms of upper or lower respiratory tract infection and had no abdominal pain, vomiting, diarrhea, dysuria, ear discharge, or recent drug exposure.

On general examination, she was conscious, rational, and had normal vital parameters. Her highest recorded temperature was 40.2°C. Her hemodynamic status at admission showed BP 108/72 mmHg, HR 112/min, RR 20/min, SpO_2_ 98% at room air and she remained stable with no hypotension, no signs of shock, and no respiratory distress. On inspection, the patient had facial swelling, strawberry tongue, generalized erythematous maculopapular rash all over the face, neck, and extremities. Cardiovascular and respiratory examinations were normal. Abdominal examination showed the patient had a palpable liver 6 cm below right costal margin, soft, non‐tender; spleen was palpable 3 cm below left costal margin, firm, soft, and non‐tender. Further examination revealed bilateral pitting edema and bilateral cervical and inguinal lymphadenopathy. Two days past admission, the patient developed bilateral conjunctivitis with mucopurulent discharge. The patient received maintenance fluids only; no bolus/no quota fluid was administered. Hematocrit remained stable without evidence of hemoconcentration. The chronological progression of clinical features and corresponding laboratory fluctuations are summarized in Table 1.

**TABLE 1:** Clinical timeline.

Illness day	Key clinical features	Platelets (×10^3^/μL)	AST (U/L)	ALT (U/L)	Key interventions
Day 1	Fever, rash, facial swelling	230	Normal	Normal	Admission
Day 3	Conjunctivitis, edema	120 (declining)	Rising	Rising	Echo performed
Day 4	Persistent fever	29.6 (nadir)	1510	1353	IVIG given
Day 5	Afebrile	Stabilizing	Declining	Declining	Supportive care

The constellation of fever > 5 days, mucocutaneous changes, lymphadenopathy, edema of extremities, and conjunctivitis raised strong suspicion for Kawasaki disease (KD), prompting urgent evaluation for possible KD.

### 2.1 Differential Diagnosis

- Kawasaki disease (primary consideration).
- Dengue infection with KD‐like features.
- MIS‐C.
- Viral hepatitis.
- Tuberculosis.
- Bacterial infections.

### 2.2 Laboratory Findings

Serial laboratory monitoring showed a decline in white blood cell count from 28.3 × 10^3^/μL at admission to 8.6 × 10^3^/μL over 72 h. Platelet count decreased from 230 × 10^3^/μL to a nadir of 29.6 × 10^3^/μL on Day 4, followed by gradual recovery beginning Day 5. At presentation, her initial liver enzymes were within normal limits, but subsequently liver enzymes increased markedly over the next 48–72 h (AST 1510 U/L; ALT 1353 U/L) and subsequently declined progressively over the following 5–7 days (Table 2).

**TABLE 2:** Laboratory investigations.

Test	Admission value	Follow‐up value	Reference range
WBC	28.3 × 10^3^/μL	8.6 × 10^3^/μL	4.0–11.0 × 10^3^/μL
Hemoglobin	10.8 g/dL	8.42 g/dL	11–15.5 g/dL
Hematocrit	40.6%	52.3%	35%–47%
Platelets	230 × 10^3^/μL	29.6 × 10^3^/μL (nadir)	150–400 × 10^3^/μL
AST	28 U/L	1510 U/L (peak)	8–33 U/L
ALT	33 U/L	1353 U/L (peak)	7–56 U/L
ALP	123 U/L	501 U/L	42–306 U/L
LDH	258 U/L	1191 U/L	140–300 U/L
Ferritin	111 ng/mL	650 ng/mL	13–150 ng/mL
HS‐CRP	13 mg/L	38.9 mg/L	1–5 mg/L
ESR	35 mm/h	19 mm/h	0–15 mm/h

Peripheral smear showed normocytic, normochromic features.

**FIGURE 1:**
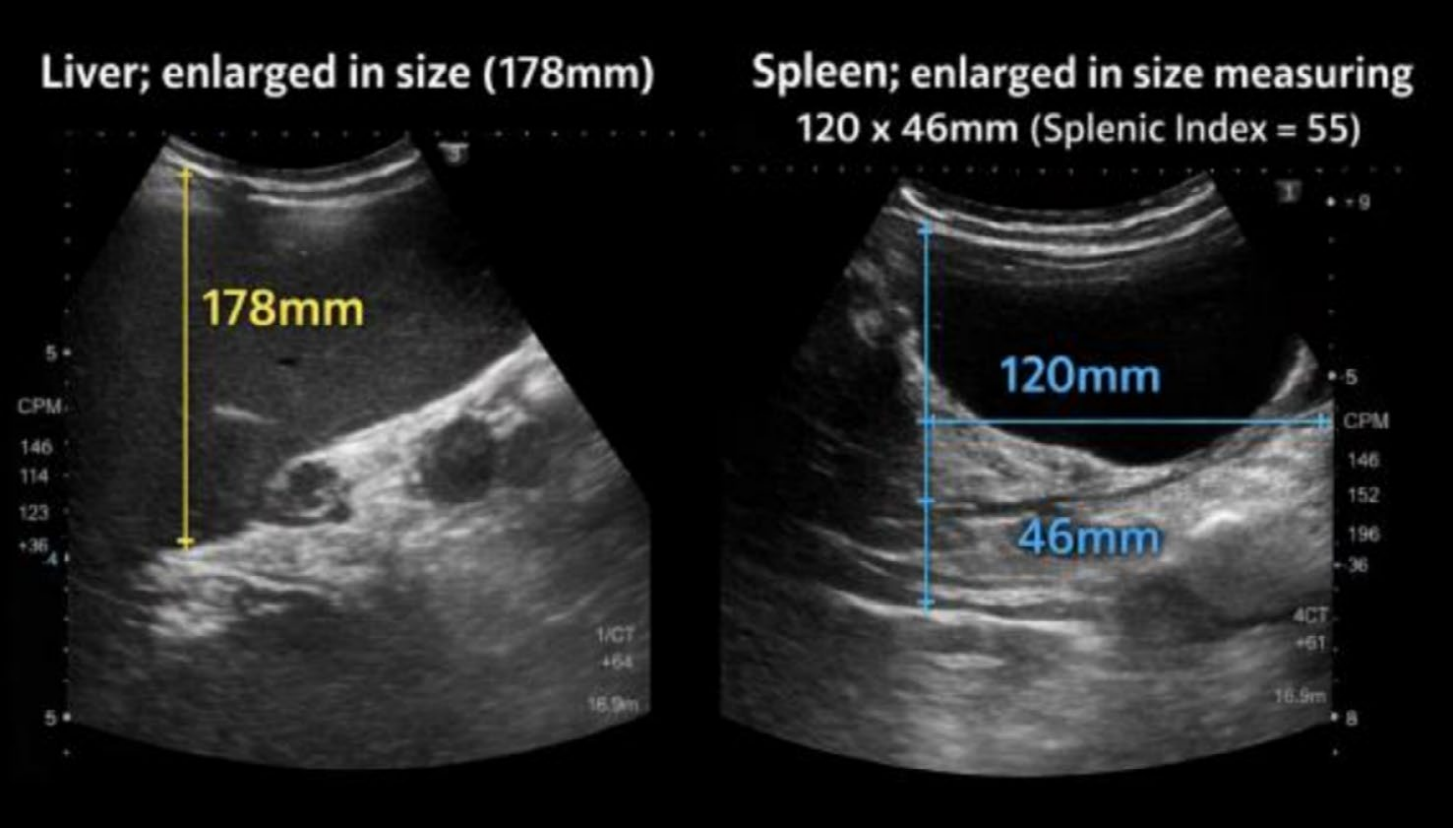
Abdominal ultrasound demonstrating hepatosplenomegaly, with enlarged liver and spleen consistent with dengue‐associated visceral involvement.

Ultrasound abdomen confirmed hepatospleenomegaly (Figure 1).

**FIGURE 2:**
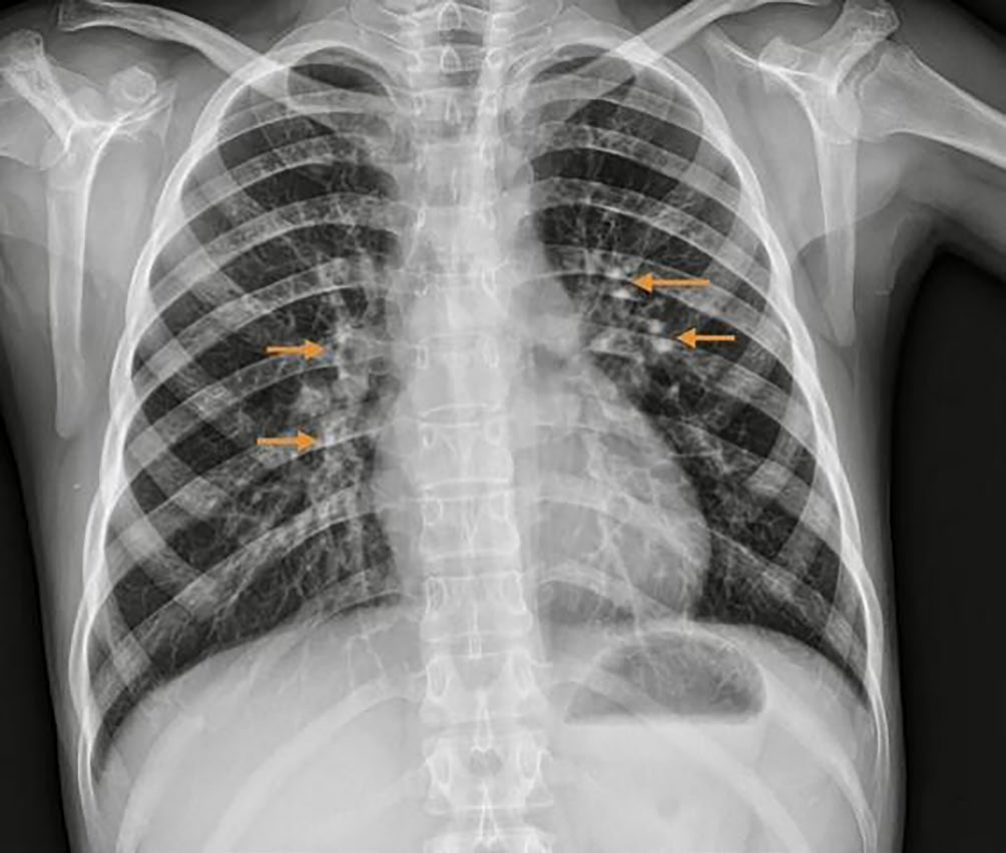
Chest X‐ray demonstrating bilateral hilar lymphadenopathy, with prominence of the hilar shadows suggestive of reactive lymph node enlargement in the context of acute systemic inflammatory illness.

CXR showed hilar lymphadenopathy (Figure 2).

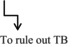

To rule out TB

- Two gastric lavage and two sputum GeneXpert samples were negative.
- No TB exposure history.

### 2.3 Dengue Evaluation

On day 2 of presentation, dengue testing showed:

- IgM: positive.
- IgG: positive.
- NS1 antigen: negative.

This profile indicated secondary dengue infection. Clinically, the patient had no evidence of plasma leakage, no hemoconcentration after fluids, and no bleeding manifestations.

An extensive evaluation was undertaken to exclude other causes since dengue rarely presents with a KD‐like phenotype.

### 2.4 Viral Hepatitis Panel

HAV IgM, HEV IgM, HBsAg, Anti‐HCV were all negative, excluding viral hepatitis as a cause of transaminitis.

### 2.5 COVID‐19/MIS‐C

- COVID PCR negative.
- D‐dimer and fibrinogen normal.
- No hypotension, shock, or cardiac dysfunction.

There was no history of SARS‐CoV‐2 infection within the preceding 8 weeks. Inflammatory profile did not demonstrate hypercoagulability, myocardial dysfunction, or shock typically seen in MIS‐C. The absence of multi‐organ involvement further reduced the likelihood of MIS‐C.

### 2.6 Bacterial Infections

- Blood cultures: negative.
- Urine culture: negative.
- Throat culture/ASO: negative.

### 2.7 Other Systems

- No joint swelling or arthritis.
- No mucosal bleeding or epistaxis.
- No meningeal signs.
- No drug exposure suggestive of hypersensitivity.

### 2.8 Cardiac Evaluation

Echocardiography was performed on illness Day 3 and demonstrated normal coronary artery dimensions for body surface area and preserved left ventricular systolic function with ejection fraction of 62%. No pericardial effusion or valvular abnormalities were noted. Troponin and BNP levels were normal.

A normal echocardiogram during the acute phase does not exclude KD, as coronary artery abnormalities may evolve during the subacute phase according to the 2024 AHA guidelines. Therefore, classical KD could not be definitively excluded at this stage.

## 3 Management and Outcome

Given the risk of delayed diagnosis leading to coronary artery aneurysms, and before dengue serology was available, IVIG 2 g/kg over 12 h was administered according to KD guidelines to prevent cardiac complications. The patient demonstrated a clear clinical response following IVIG administration, with fever settling within 14 h of completing the infusion. Her rash began to fade within the first 24 h, conjunctival discharge decreased significantly by 48 h, and peripheral edema resolved over the next 2–3 days. Laboratory markers also improved, with platelet counts stabilizing by Day 4 and liver enzymes beginning to decline within 72 h. High‐dose aspirin was deferred due to progressive thrombocytopenia and risk of bleeding. Low‐dose antiplatelet therapy was not initiated as platelet counts improved rapidly and no coronary abnormalities were detected. This represents a deviation from standard AHA KD guidelines and was guided by bleeding risk in the setting of confirmed dengue infection. The patient was followed up at 2 weeks post‐discharge. Repeat echocardiography at 6 weeks demonstrated normal coronary artery dimensions with no evidence of aneurysm or ectasia. She remained afebrile with complete resolution of clinical symptoms. Supportive dengue management was provided, including careful hydration, antipyretics, and serial hematologic and hepatic monitoring. Platelets initially fell but stabilized over subsequent days. Liver enzymes gradually normalized. She remained hemodynamically stable throughout her admission. No coronary involvement, shock, desquamation, or bleeding complications occurred. She was discharged in improved condition.

## 4 Conclusion and Results (Outcome and Follow‐Up)

The patient showed a rapid clinical response following IVIG:

- Fever resolved within 14 h.
- Rash improved within 24 h.
- Conjunctivitis improved within 48 h.
- Edema resolved over 2–3 days.

Laboratory trends:

- Platelets stabilized after Day 4.
- Liver enzymes declined within 72 h.

She remained hemodynamically stable with:

- No bleeding.
- No shock.
- No coronary involvement.

Follow‐up:

- 2‐week clinical review: complete symptom resolution.
- 6‐week echocardiography: normal coronary arteries.

She was discharged in stable condition with full recovery.

## 5 Discussion

Although dengue and Kawasaki disease (KD) are distinct, recent reports document that dengue can present with KD‐like vasculitic features in children. Several case series note prolonged fever, conjunctival injection, mucosal inflammation (cracked lips), and even coronary artery involvement in dengue patients, mimicking KD [4]. Conjunctivitis in classical KD is typically bilateral and non‐suppurative; however, in overlapping infectious inflammatory states such as dengue, mucopurulent discharge may be observed, potentially contributing to diagnostic ambiguity. No single test distinguishes KD from dengue, but a combination of serology and clinical clues can help. Dengue can be confirmed by detecting viral antigen or antibodies (e.g., NS1 antigen, IgM) on rapid tests, which are affordable and recommended in low‐resource settings. By contrast, KD has no specific pathogen, so diagnosis relies on clinical criteria and nonspecific labs. Inflammatory markers tend to differ: acute KD typically causes very high CRP and IL‐6–driven megakaryocyte activation causing thrombocytosis (platelet count > 500 × 10^3^/mm^3^) in the subacute phase, whereas dengue usually shows leukopenia and progressive thrombocytopenia. Along with nonstructural protein NS1 induced endothelial injury and a cytokine storm (TNF‐α, IL‐6, IFN‐γ) leading to capillary leak which are not seen in KD [5].

Our patient's age (12 years) is atypical for classical KD, which most commonly affects children under 5 years. Adolescent presentations are less frequent but have been reported, often associated with higher inflammatory markers and increased risk of delayed diagnosis. The age factor in this case further complicated differentiation between primary KD and infection‐triggered inflammatory mimicry [6].

IVIG has no proven role in pure dengue and is expensive, so its use is limited to KD‐like cases. There is no consensus, but the balance of experience suggests that IVIG can be beneficial in dengue‐triggered KD (or “KD‐like”) syndromes, despite some uncertainty. Clinicians are advised to maintain a high index of suspicion: if a dengue patient has persistent fever or evolving mucocutaneous features, consider KD and treat with IVIG to avert complications [7] as its administration can lead to transient clinical improvement because of its broad immunomodulatory effects. According to the 2024 AHA guidelines, IVIG should not be delayed in patients fulfilling clinical KD criteria even if initial echocardiography is normal, as coronary abnormalities may develop in the subacute phase. IVIG downregulates pathogenic cytokine release, neutralizes circulating immune complexes, and suppresses excessive T‐cell activation; mechanisms that overlap with the immune‐driven vasculitic phase seen in dengue.

In resource‐constrained environments where confirmatory testing may be delayed or limited, this overlap between dengue and KD creates a significant diagnostic challenge, reinforcing the importance of pragmatic algorithms and early treatment decisions based on evolving clinical features.

## Author Contributions

**Saad Bin Toufeeq:** conceptualization, writing – original draft. **Ammara Arshad:** formal analysis, investigation, methodology. **Ali Bin Toufeeq:** data curation. **Ahmad Sama:** investigation, validation. **Neykzad Wadeer:** project administration, resources, validation. **Nabeel Ahmad:** supervision, writing – review and editing.

## Funding

This case report received no external funding. All investigations and care were part of routine clinical management.

## Ethics Statement

Ethical approval was not required for this single‐patient case report in accordance with institutional guidelines. All procedures adhered to ethical clinical standards.

## Consent

Written informed consent was obtained from the patient's legal guardian for publication of this case report and accompanying images.

## Conflicts of Interest

The authors declare no conflicts of interest.

## Data Availability

The data supporting the findings of this case report are available from the corresponding author upon reasonable request, subject to ethical and privacy considerations.

## References

[1] M. B. Khan , Z.‐S. Yang , C.‐Y. Lin , et al., “Dengue Overview: An Updated Systemic Review,” Journal of Infection and Public Health 16, no. 10 (2023): 1625–1642. 37595484 10.1016/j.jiph.2023.08.001

[2] S. Sopontammarak , W. Promphan , S. Roymanee , and S. Phetpisan , “Positive Serology for Dengue Viral Infection in Pediatric Patients With Kawasaki Disease in Southern Thailand,” Circulation Journal 72, no. 9 (2008): 1492–14944, 10.1253/circj.cj-08-0158. 18724028

[3] S. Bittmann , E. Luchter , L. Bittmann , and E. Moschüring‐Alieva , “The Link Between Dengue Fever and Kawasaki Disease in Children,” Asian Journal of Pediatric Research 14, no. 10 (2024): 22–28.

[4] C. Govardhan , B. Narayanaswamy , P. Ragavaiah Naidu , and F. D'Souza , “Concomitant Dengue Fever and Kawasaki Disease in an Infant: Case Report and Review of the Literature,” Paediatrics and International Child Health 25 (2025): 1–5. 10.1080/20469047.2025.2561344 41020614

[5] A. Agrafiotou , E. Sapountzi , A. Margoni , and L. Fotis , “Immunophenotype of Kawasaki Disease: Insights Into Pathogenesis and Treatment Response,” Life 15, no. 7 (2025): 1012, 10.3390/life15071012. 40724515 PMC12299537

[6] C. Manlhiot , R. S. M. Yeung , N. A. Clarizia , N. Chahal , and B. W. McCrindle , “Kawasaki Disease at the Extremes of the Age Spectrum,” Pediatrics 124, no. 3 (2009): e410–e415, 10.1542/peds.2009-0099. 19706564

[7] Y. Sidharth , “Profile of Children With Kawasaki Disease Associated With Tropical Infections,” Indian Journal of Pediatrics 89 (2021): 759–764, 10.1007/S12098-021-03. 34935098 PMC8691965

